# Bovine Herpesvirus-4-Based Vector Delivering Peste des Petits Ruminants Virus Hemagglutinin ORF Induces both Neutralizing Antibodies and Cytotoxic T Cell Responses

**DOI:** 10.3389/fimmu.2018.00421

**Published:** 2018-03-05

**Authors:** Francesca Macchi, José Manuel Rojas, Andrea Elizabeth Verna, Noemí Sevilla, Valentina Franceschi, Giulia Tebaldi, Sandro Cavirani, Verónica Martín, Gaetano Donofrio

**Affiliations:** ^1^Department of Medical Veterinary Science, University of Parma, Parma, Italy; ^2^Centro de Investigación en Sanidad Animal (CISA-INIA), Instituto Nacional de Investigación y Tecnología Agraria y Alimentaria, Madrid, Spain

**Keywords:** BoHV-4, PPRV, DIVA vaccines, H antigen, viral vaccines

## Abstract

Peste des Petits Ruminants Virus (PPRV) is an extremely infective morbillivirus that primarily affects goats and sheep. In underdeveloped countries where livestock are the main economical resource, PPRV causes considerable economic losses. Protective live attenuated vaccines are currently available but they induce antibody responses similar to those produced in PPRV naturally infected animals. Effective vaccines able to distinguish between vaccinated and naturally infected animals are required to PPRV control and eradication programs. Hemagglutinin (H) is a highly immunogenic PPRV envelope glycoprotein displaying both hemagglutinin and neuraminidase activities, playing a crucial role in virus attachment and penetration. In this study, a recombinant Bovine Herpesvirus-4 (BoHV-4)-based vector delivering an optimized PPRV-Hemagglutinin expression cassette, BoHV-4-A-PPRV-H-ΔTK, was assessed in immunocompetent C57BL/6 mice. BoHV-4-A-PPRV-H-ΔTK-immunization elicited both cellular and humoral immune responses with specific T cell, cytotoxic T lymphocyte, and sero-neutralizing antibody against PPRV. These data suggest recombinant BoHV-4-A-PPRV-H-ΔTK as an effective vaccine candidate to protect against PPRV herd infection and potentially applicable for eradication programs.

## Introduction

Peste des Petits Ruminants (PPR) is an often fatal, highly contagious, and devastating disease affecting domestic small ruminants and especially goats. PPR is an Office International des Epizooties (OIE)-listed disease (http://www.oie.int/animal-health-in-the-world/oie-listed-diseases-2018/), endemic in several countries such as India, Turkey, Africa, and Southwest and Central Asia. Mouth and tongue lesions, cough, diarrhea, nasal and ocular discharge, and depression are typical clinical PPR disease signs. The PPR disease etiological agent is Peste des Petits Ruminants Virus (PPRV), a single-stranded negative sense enveloped RNA virus belonging to *Paramixoviridae* family, *Morbillivirus* genus (1) whose genome contains six genes coding for eight proteins. Among these, Hemagglutinin (H) is a structural glycoprotein with hemagglutinin and neuraminidase activities, involved in host cell targeting and virus attachment. H glycoprotein is an immunodominant antigen which, alone, can stimulate a protective immune-response when delivered by several viral vectors, mainly based on adenovirus (2–4) and poxvirus (5, 6). These antigen immune-properties would allow the generation of a Differentiating Infected from Vaccinated Animals (DIVA) vaccine. Since PPRV H glycoprotein is the only PPRV antigen expressed by the viral vector, the use of an ELISA against a different antigen, such as PPRV nucleo-capsid protein (N), would allow to distinguish naturally infected animals from vaccinated animals. Viral vectors are not only simply delivery systems but they can also work as adjuvants, unspecificaly stimulating the immune system and therefore increasing the specific active/protective immunity. Different classes of viruses have been tested as viral vectors and each presents particular advantages and disadvantages, depending on their biological characteristics and on the host, who needs to be protected toward a specific disease. Hence, it is arduous to predict which viral vector could be the best. A specific viral-vector should be able to confer selective immunization only against a specific pathogen and not toward others. Consequently, it would be of great interest to explore new vector vaccines based on different viruses. *Bovine herpesvirus 4* (BoHV-4) is a dsDNA genome virus belonging to *Herpesviridae* family, *Gammaherpesvirus* sub-family and *Rhadinovirus* genus. BoHV-4 natural host is cattle, whereas its best experimental host is the rabbit. However, BoHV-4 has been isolated from domestic and non-domestic bovine species such as African buffalo (*Syncerus caffer*) (7), American bison (*Bison bison*), or zebus (*Bos indicus*) and small ruminants such as sheep and goats (8). Some feline isolates from lions (9) and cats (10) were also reported. Moreover, BoHV-4 isolates were also obtained from the kidney of an apparently healthy monkey (*Aotus trivirgatus*) (11). BoHV-4 can replicate *in vitro* in primary cultures and cell lines from a variety of animal species (12–18), whereas *in vivo*, it can experimentally infect mice (16, 19, 20), rats (21), rabbits (15), sheep (13), swine (22), and goats (18). Moreover, *ex vivo* non-human primate tissue explants infections have also been observed (paper in preparation). Another BoHV-4 important feature, which makes it an attractive gene delivery vector, is that in contrast to other gamma herpesviruses, BoHV-4 is not oncogenic and its infection is not directly linked to a specific pathology. Since BoHV-4-based vector has been successfully employed to immunize mice (16, 19, 20), sheep (13), and goats (18), in the present work, an exploratory immunization study for PPRV in mice, before applying BoHV-4-based vector in sheep and goats, was performed. A recombinant BoHV-4 expressing the PPRV Hemagglutinin gene (Nigeria 75/1 strain) was generated. BoHV-4-A-PPRV-H-ΔTK immunized mice developed both PPRV neutralizing antibodies and PPRV specific T-cell responses. These data indicate that this BoHV-4-based vector could be an effective PPR vaccine candidate for small ruminants that could distinguish between infected and vaccinated animals.

## Materials and Methods

### Cells and Viruses

In this study, HEK (Human Embryo Kidney) 293 T (ATCC: CRL-11268), BEK (Bovine Embryo Kidney) from Dr. M.Ferrari, Istituto Zooprofilattico Sperimentale, Brescia, Italy (BS CL-94), and BEK *cre*, expressing *cre* recombinase (14), were cultured in Eagle’s Minimal Essential Medium (EMEM, Gibco) containing 10% fetal bovine serum (FBS), 2 mM of L-glutamine (Gibco), 100 IU/ml of penicillin (Gibco), 100 µg/ml of streptomycin (SIGMA), and 0.25 µg/ml of amphotericin B (Gibco) and were incubated at 37°C, 5% CO_2_ in a humidified incubator. Vero Dog-SLAM (VDS) and RMA-s cell lines (23), kindly provided by Dr. Parida (IAH, Pirbright, UK) and Dr McArdle (The Nottingham Trent University, UK), respectively, were cultured as described in Ref. (24, 25).

### Constructs Generation

Synthetic PPRV-H ORF was first amplified from pGEM-T Easy-PPRV-H template by PCR using NheI-PPRV-H sense (5′-ccccgctagcccaccatgtccgcacaaagggaaagg-3′) and Phos-PPRV-H antisense (5′-agactggattacatgttacctc-3′) pair of primers in order to insert NheI restriction site at 5′ terminus and a phosphate group at 3′ terminus. The PPRV-H amplicon generated was then cloned into NheI/SalI blunt cut pIgK-E_2_BVDV_3-_gD_106_ intermediate shuttle vector (Clontech) to generate pIgK-PPRV-H-gD_106_. The gD_106_ tagged fragment was excised from the intermediate plasmid cutting with NheI and BamHI blunt restriction enzymes to be subsequently cloned inside the pINT_2_-EGFP final shuttle vector cut with NheI and SmaI restriction enzymes in order to generate pINT_2_-PPRV-H-gD_106_.

### Transient Transfection

HEK 293 T cells were seeded into six well plates (3 × 10^5^ cells/well) and incubated at 37°C with 5% CO_2_. When cells were sub-confluent, the culture medium was removed and the cells were transfected with pIgK-PPRV-H-gD_106_, pINT_2_-PPRV-H-gD_106_, and pEGFP-C1 using Polyethylenimine (PEI) transfection reagent (Polysciences, Inc.). Briefly, 3 µg of DNA were mixed with 7.5 µg PEI (1 mg/ml) (ratio 1:2.5 DNA:PEI) in 200 µl of Dulbecco’s modified essential medium (DMEM) high glucose (Euroclone) without serum. After 15 min at room temperature, 800 µl of medium without serum were added, and the transfection solution was transferred to the cells (monolayer) and left for 6 h at 37°C with 5% CO_2_, in a humidified incubator. The transfection mixture was then replaced with fresh medium EMEM, with 10% FBS, 100 IU/ml of penicillin, 100 µg/ml of streptomycin, and 0.25 µg/ml of amphotericin B, and incubated for 24 h at 37°C with 5% CO_2_.

### Western Immunoblotting

Protein cell extracts were obtained from a six-well confluent plate of HEK 293 T cells transfected with pIgK-PPRV-H-gD_106_, pINT_2_-PPRV-H-gD_106_, and pEGFP-C1 and from 25-cm^2^ confluent flasks of BEK cells infected with BoHV-4-A-PPRV-H-ΔTK by adding 100 µl of cell extraction buffer (50 mM Tris–HCl, 150 mM NaCl, and 1% NP-40; pH 8). To analyze cell extracts, a 10% SDS-PAGE gel electrophoresis was used. After protein transfer in PVDF membranes by electroblotting, the membranes were incubated with primary bovine anti gD_106_ monoclonal antibody (clone 1B8-F11; VRMD, Inc., Pullman, WA, USA) diluted 1:10,000 and then probed with horseradish peroxidase-labeled anti-mouse immunoglobulin (SIGMA), diluted 1:10,000, and bands visualized by enhanced chemiluminescence (ECL KIT; PIERCE).

### BAC Recombineering and Selection

Recombineering was performed as previously described (26) with some modifications. For heat-inducible homolog recombination in SW102 *Escherichia coli* (*E. coli*), containing the BAC-BoHV-4-A-TK-KanaGalK-TK genome targeted into the TK locus with KanaGalK selector cassette, the PvuI linearized pTK-CMV-PPRV-H-TK expression cassette was used. After recombineering, only those colonies that were kanamycin negative and chloramphenicol positive were kept and grown overnight in 5 ml of LB containing 12.5 mg/ml of chloramphenicol. BAC-DNA was purified and analyzed through HindIII restriction enzyme digestion. DNA was separated by electrophoresis in a 1% agarose gel, stained with ethidium bromide, and visualized through UV light. Original detailed protocols for recombineering can also be found at the recombineering website (https://redrecombineering.ncifcrf.gov/).

### Southern Blotting

To further confirm our results, a Southern Blotting with a probe spanning H sequence was performed. DNA from 1% agarose gel was capillary transferred to a positively charged nylon membrane (ROCHE) and cross-linked by UV irradiation by standard procedures (14). The membrane was pre-hybridized in 50 ml of hybridization solution (7% SDS, 0.5 M phosphate, pH 7.2) for 1 h at 65°C in a rotating hybridization oven (Techna Instruments).

H probe labeled with digoxigenin was generated by PCR with NheI-PPRV-H sense (5′- ccccgctagcccaccatgtccgcacaaagggaaagg -3′) and Phos-PPRV-H antisense (5′- agactggattacatgttacctc -3′) primers, as previously described (15). The PCR amplification reaction was carried out in a final volume of 50 µl, containing 10 mmol Tris–hydrochloride pH 8.3, 5% Dimethyl Sulfoxide (DMSO), 0.2 mmol deoxynucleotide triphosphates, 2.5 mM MgSO_4_, 50 mM KCl, and 0.25 µM of each primer. One hundred nanograms of DNA were amplified over 35 cycles, each cycle consisting of 1 min of denaturation at 94°C, 1 min of primer annealing at 60°C, and 2 mins of chain elongation with 1U of Taq DNA polymerase (Fermentas) in addition to 1 µl of Digoxigenin-11-dUTP, alkali-labile (Roche Life Science) at 72°C.

### Cell Culture Electroporation and Recombinant Virus Reconstitution

BEK or BEK *cre* cells were maintained as a monolayer with complete DMEM growth medium with 10% FBS, 2 mM l-glutamine, 100 IU/ml penicillin and 100 µg/ml streptomycin. When cells were sub-confluent (70–90%) they were split to a fresh culture flask (i.e., every 3–5 days) and were incubated at 37°C in a humidified atmosphere of 95% air, 5% CO_2_. BAC-DNA (5 µg) was electroporated in 600 µl DMEM without serum (Equibio Apparatus, 270 V, 960 mF, 4-mm gap cuvettes) into BEK and BEK *cre* cells from a confluent 25-cm^2^ flask. Electroporated cells were returned to the flask, after 24 h the medium was replaced with fresh medium, and cells were split 1:2 when they reached confluence at 2 days post-electroporation. Cells were left to grow until the appearance of cytopathic effect (CPE).

### Viruses and Viral Replication

BoHV-4-A-PPRV-H-ΔTK and BoHV-4-A were propagated by infecting confluent monolayers of BEK cells at a multiplicity of infection (MOI) of 0.5 tissue culture infectious doses 50 (TCID50) per cell and maintained in medium with only 2% FBS for 2 h. The medium was then removed and replaced with fresh EMEM containing 10% FBS. When CPE affected the majority of the cell monolayer (~72 h post infection), the virus was prepared by freezing and thawing cells three times and pelleting the virions through a 30% sucrose cushion, as previously described (27). Virus pellets were then resuspended in cold EMEM without FBS. TCID50 were determined on BEK cells by limiting dilution.

### Viral Growth Curves

BEK cells were infected with BoHV-4-A and BoHV-4-A-PPRV-H-ΔTK at a M.O.I. of 0.1 TCID50/cell and incubated at 37°C for 4 h. Infected cells were washed with serum-free EMEM and then overlaid with EMEM containing 10% FBS, 2 mM L-glutamine, 100 IU/ml penicillin, 100 mg/ml streptomycin, and 2.5 mg/ml Amphotericin B. The supernatants of infected cultures were harvested after 24, 48, 72, and 96 h, and the amount of infectious virus was determined by limiting dilution on BEK cells. Viral titer differences between each time point are the averages of triplicate measurements ± standard errors of the mean (*p* > 0.05 for all time points as measured by Student’s *t*-test).

### Animals and Immunizations

Seven- to eight-week old female C57BL/6 mice (Harlan) animals were inoculated and boosted after 21 days intraperitoneally (ip) with PBS (group 1; *n* = 10), with 10^6^ TCID_50_/ml of BoHV-4-A (group 2; *n* = 10) or with 10^6^ TCID_50_/ml of BoHV-4-A-PPRV-H-ΔTK (group 3; *n* = 10). Animals were bled at 14, 28, and 36 days post first immunization. Five animals per group were sacrificed at day 7 post-boost to perform T cell response experiments. All animal experiments were performed in a disease-secure isolation facility (BSL3) at the CISA (INIA) in strict accordance with the recommendations of the Code for Methods and Welfare Considerations in Behavioral Research with Animals (Directive 867609EC; RD1201/2005).

### Flow Cytometry Intracellular Cytokine Staining Assays

Splenocytes from inoculated mice were prepared as previously described (24). For responses to PPRV, splenocytes were cultured overnight with BEI-inactivated PPRV (Nig’75) (28). To assess responses to PPRV-H murine T cell epitopes H5 (H(551-559) YFYPVRLNF) and H9 (H(427-441) ITSVFGPLIPHLSGM) (29), splenocytes were expanded *in vitro* for 1 week with 10 µg/ml peptide before measuring IFN-γ responses. For intracellular IFN-γ measurements, cells were cultured at 10^6^ cells per well in the presence of different stimuli (peptide or PPRV) overnight before the addition of 10 µg/ml brefeldin-A (Sigma) for the last 5 h of incubation. Phorbol myristyl acetate (20 ng/ml) and ionomycin (1 µg/ml) (both from sigma) stimulation was used as positive control for IFN-γ production. Vehicle (DMSO)-stimulated (no peptide) or irrelevant peptides (gp33-41 peptide (KAVYNFATC) from lymphocytic choriomeningitis virus) were used as negative control. No differences in background IFN-γ production was detected between these negative control groups. Following stimulation, cells were stained with anti-mouse CD4-FITC and anti-mouse CD8-PerCP antibodies (BDpharmingen). Cells were fixed and permeabilized in PBS containing 4% paraformaldehyde and 0.1% saponin (wt/vol). Cells were then stained with anti-mouse IFN-γ-PE (BD pharmingen) and acquired using a FACSCalibur flow cytometer (Becton Dickinson). Gating strategy is described in Ref. (29). Gating for positive IFN-γ positive events was set using isotype and fluorescence minus one channel controls. Data were analyzed with FlowJo software (TreeStar Inc.).

### Flow Cytometry Cytotoxicity Assays

Splenocytes from BoHV-4-A-PPRV-H-ΔTK immunized mice were expanded with H5 peptide for 1 week *in vitro*. These stimulated splenocytes were used as effector cells. RMA/s target cells were labeled with PKH67 green fluorescent linker as described in Ref. (30) and pulsed with relevant peptide. Vehicle-pulsed (no peptide) RMA/s cells were used as negative control. Effector cells and target cells were incubated for 4 hours at 37°C in 96 U-bottom well plates. Cells were then transferred to FACS tubes, dead cells labeled with propidium iodide (PI) (2 µg/ml), and samples immediately analyzed by flow cytometry. Target cells were gated on bright FL1+ cells. Positive maximum cell death controls (target cells in PBS + 0.2% saponin) and spontaneous cell death controls were used in all experiments. The percentage of specific target cell lysis was calculated following the formula: % specific lysis = 100 × (% PI+ target – % spontaneous death)/(% maximum death − % spontaneous death).

### PPRV Neutralization Assays

Serum samples were inactivated for 30 min at 56°C and tested for the presence of neutralizing antibodies as previously described (31). Briefly, Nigeria 75/1 PPRV stock was incubated with serial dilutions of inactivated sheep serum for 1 hour at RT in triplicate. VDS cells at a concentration of 1.5 × 10^5^ cells/ml were added to each well and incubated for 7 days, fixed with 2% formaldehyde and cells visualized by crystal violet staining. Wells without virus served as controls. The plates were monitored for PPRV CPE for 7 days. The VNT titer was defined as the highest dilution of serum that inhibited 50% of the CPE. Sera with VNT titers of 1:10 were considered negative.

### Statistical Analysis

Power analysis (32) was used to determine treatment group size to assess T cell responses and PPRV seroneutralization. Statistical analysis was performed using Prism 5.0 software (Graphpad Software Inc., USA). Mann–Whitney test was used to compare IFN-γ production in CD4+ and CD8+ T cells. Levels of significance were *p < 0.05, **p < 0.01, and ***p < 0.001.

## Results

### BoHV-4-A-PPRV-H-ΔTK Generation

**Figure 1:**
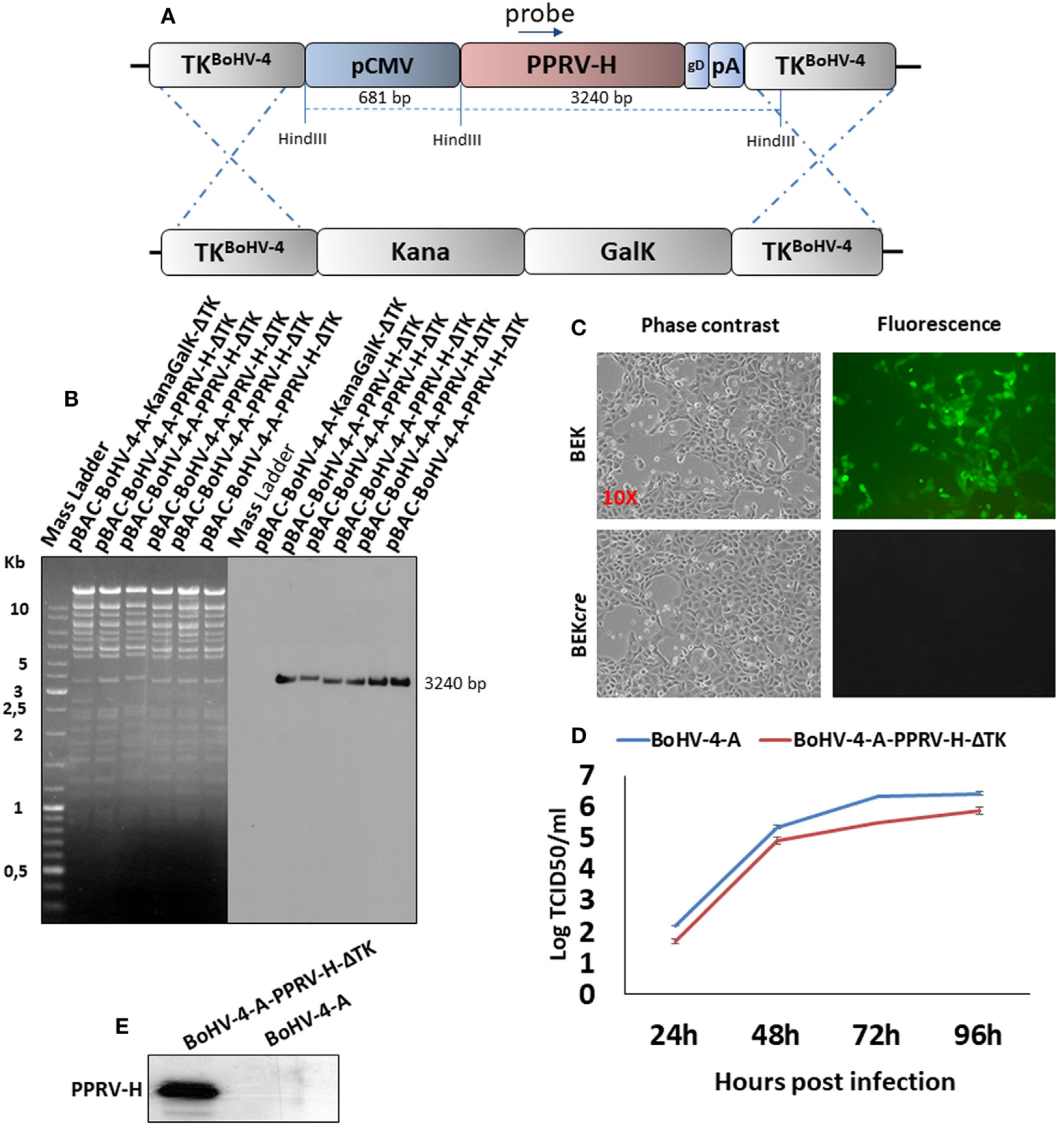
Diagram (not to scale); **(A)** summarizing the heat-inducible homologous recombination in SW102 containing pBAC-BoHV-4-A-TK-KanaGalK-TK, where the Kana/GalK cassette was replaced with the CMV-PPRV-HgD_106_ expression cassette flanked by bovine herpesvirus 4 (BoHV-4) TK sequences, located in pINT2 shuttle plasmid vector. **(B)** Representative 2-deoxy-galactose resistant colonies tested by HindIII restriction enzyme analysis, agar gel electrophoresis, and Southern blotting performed with specific probes for the peste des petits ruminants virus hemagglutinin (PPRV-H) ORF ORFs. The 2,650 bp band, corresponding to the un-retargeted pBAC-BoHV-4-A-TK-KanaGalK-TK control, has been replaced by a 3240 bp band in pBAC-BoHV-4-A-CMV-PPRV-H-ΔTK. **(C)** Representative phase contrast microscopic images of plaque formed by viable reconstituted recombinant BoHV-4-A-PPRV-H-ΔTK after the corresponding bacterial artificial chromosome (BAC) DNA electroporation into bovine embryo kidney (BEK) cells expressing *cre* recombinase (Magnification, ×10). **(D)** Replication kinetics of BoHV-4-A-PPRV-H-ΔTK growth on BEK cells and compared with the parental BoHV-4-A isolate. The data presented are the mean ± standard errors of triplicate measurements (*P* > 0.05 for all time points as measured by Student’s *t*-test). **(E)** Western immunoblotting of cells, infected with BoHV-4-A-PPRV-H-ΔTK or the parental BoHV-4-A used as a negative control. The lanes were loaded with different amounts of total protein cell extract (5, 10, and 20 µg).

Based on the assumption that PPRV-H antigen could induce a protective immune response, a recombinant BoHV-4, BoHV-4-A-PPRV-H-ΔTK, delivering an optimized CMV-PPRV-HgD_106_ expression cassette (Figure 1A), was generated by heat inducible homologous recombination in SW102 *E. Coli* strain containing pBAC-BoHV-4-A-KanaGalK-ΔTK (14) (Figure 1B). The so obtained pBAC-BoHV-4-A-PPRV-H-ΔTK recombinant viral genome authenticity was first assessed by HindIII restriction enzyme analysis and then confirmed by Southern Blotting using a PPRV-H specific probe. Clonal stability was ascertained by growing the positive clone over 20 passages. Recombinant BoHV-4-A-PPRV-H-ΔTK infectious viral particles were then obtained electroporating BEK or BEK*cre* cells. These last cells allowed the depletion of the BAC/GFP cassette from the recombinant viral genome, as shown by the loss of green plaques (Figure 1C). Furthermore, BoHV-4-A-PPRV-H-ΔTK showed no replication defects comparing with the BoHV-4-A parental strain (Figure 1D) and expressed PPRV-H protein (Figure 1E).

### BoHV-4-A-PPRV-H-ΔTK Immunization Induces T Cell Response against PPRV

**Figure 2:**
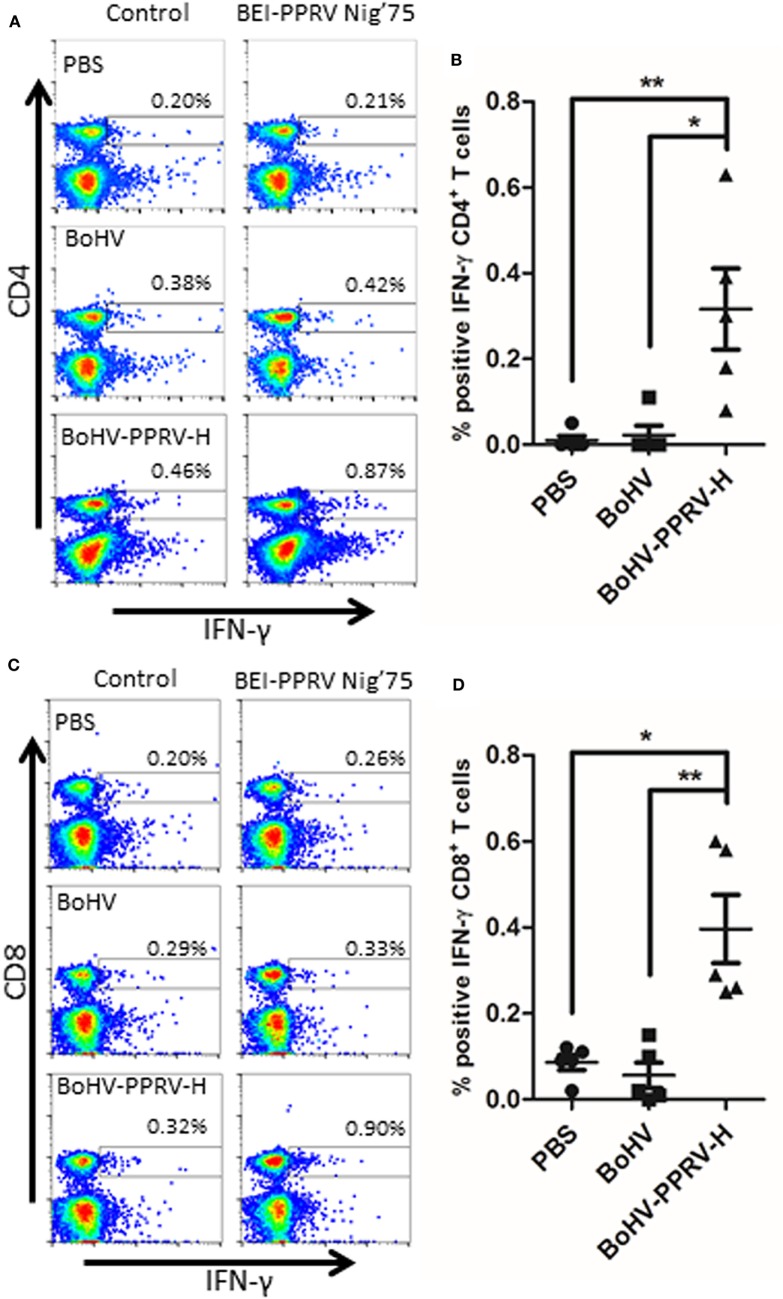
Induction of CD4+ and CD8+ T cell responses to peste des petits ruminants virus (PPRV) by BoHV-4-A-PPRV-H-ΔTK recombinant vaccine Splenocytes from vaccinated C57BL/6 mice were extracted 7 days after booster vaccination and stimulated with inactivated PPRV Nig’75 strain (BEI-PPRV Nig’75) overnight. IFN-γ production in CD4+ and CD8+ T cells was assessed by flow cytometry using intracellular staining. **(A)** Representative dot-plots for IFN-γ production by CD4+ T cells in mice vaccinated with PBS, bovine herpesvirus 4 [BoHV-4 (BoHV)], or BoHV-4-A-PPRV-H-ΔTK (BoHV-PPRV-H) are shown. **(B)** The average ± SD percentage of IFN-γ-producing CD4+ T cells above control in 5 mice per group at 7 days post-booster vaccination are shown. Mann–Whitney test (BoHV-4-A-PPRV-H-ΔTK vs BoHV-4-A or PBS); *p < 0.05; **p < 0.01. **(C)** Representative dot-plots for IFN-γ production by CD8+ T cells in mice vaccinated with PBS, BoHV-4-A, or BoHV-4-A-PPRV-H-ΔTK are shown. **(D)** The average ± SD percentage of IFN-γ-producing CD8+ T cells above spontaneous IFN- γ release (control) in 5 mice per group at 7 days post-booster vaccination are shown. Mann–Whitney test (BoHV-4-A-PPRV-H-ΔTK vs BoHV-4-A or PBS); *p < 0.05; **p < 0.01.

Splenocytes from C57BL/6 mice were extracted 7 days after booster immunization and IFN-γ production by T cells to inactivated PPRV Nig’75 strain was measured by flow cytometry (Figure 2). CD4+ T cells from BoHV-4-A-PPRV-H-ΔTK immunized mice produced IFN-γ in response to inactivated PPRV stimulation (Figures 2A,B). No specific IFN-γ production to PPRV was detected in CD4+ T cells from PBS- or BoHV-4-A immunized mice. Similarly, CD8+ T cells from BoHV-4-A-PPRV-H-ΔTK immunized mice produced IFN-γ in the presence of PPRV, whereas CD8+ T cells from PBS- or BoHV-4-A-immunized animals did not. These data indicate that BoHV-4-A-PPRV-H-ΔTK immunization can elicit CD4+ and CD8+ T cell responses to PPRV infection.

### BoHV-4-A-PPRV-H-ΔTK Immunization Induces Responses to PPRV-H T Cell Epitopes

**Figure 3:**
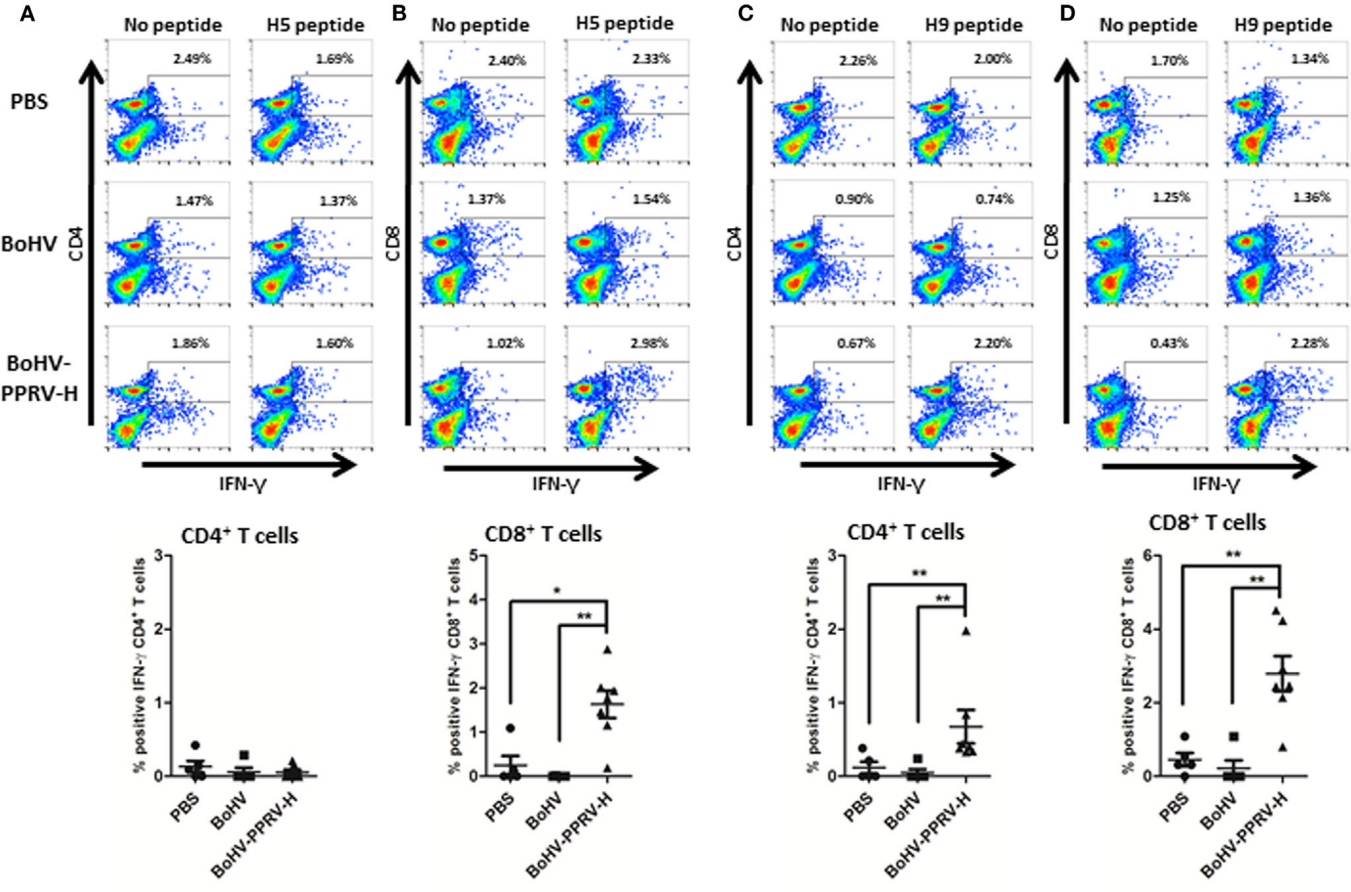
BoHV-4-A-PPRV-H-ΔTK immunization induces CD4+ and CD8+ T cell responses specific for peste des petits ruminants virus hemagglutinin (PPRV-H) epitopes Splenocytes from C57BL/6 mice immunized with PBS, BoHV-4-A (BoHV), or BoHV-4-A-PPRV-H-ΔTK (BoHV-PPRV-H) were stimulated *in vitro* with H5 or H9 peptides from PPRV-H for 1 week. IFN-γ production in CD4+ and CD8+ T cells was measured by flow cytometry using intracellular staining. Representative dot-plots for IFN-γ production to H5 peptide in **(A)** CD4+ and **(B)** CD8+ T cells in immunized mice are shown. Average ± SD specific IFN-γ production to H5 peptide in immunized mice groups (*n* = *5* for PBS and BoHV-4-A; *n* = *7* for BoHV-4-A-PPRV-H**-Δ**TK) are plotted. Mann–Whitney test [BoHV-4-A-PPRV-H**-Δ**TK (BoHV-4-A-PPRV-H) vs PBS or BoHV-4-A (BoHV)]; *p < 0.05; **p < 0.01. Representative dot-plots for IFN-γ production to H9 peptide in **(C)** CD4+ and **(D)** CD8+ T cells in immunized mice are shown. Average ± SD specific IFN-γ production to H9 peptide in immunized mice groups (*n* = *5* for PBS and BoHV-4-A; *n* = *7* for BoHV-4-A-PPRV-H**-Δ**TK) are plotted. Mann–Whitney test (BoHV-4-A-PPRV-H**-Δ**TK vs PBS or BoHV-4-A); **p < 0.01.

Several murine T cell epitopes from PPRV-H in the H-2^b^ context were previously defined (29). In the present work, H5 and H9 peptides from PPRV-H protein were used to further characterize the T cell response against PPRV after BoHV-4-A-PPRV-H-ΔTK immunization. Splenocytes from immunized mice were stimulated 1 week *in vitro* with H5 and H9 peptides and IFN-γ production was assessed by flow cytometry using intracellular staining (Figure 3). H5 peptide induced specific IFN-γ production in CD8+ T cells but not in CD4+ T cells of BoHV-4-A-PPRV-H-ΔTK-immunized mice (Figures 3A,B). No specific H5 peptide-stimulated IFN-γ production was detected in PBS or BoHV-4-A immunized mice splenocytes. H9 peptide induced specific IFN-γ production both in CD4+ and CD8+ T cells of BoHV-4-A-PPRV-H-ΔTK-immunized mice (Figures 3C,D). No H9 peptide-stimulated IFN-γ secretion was detected in PBS or BoHV-4-A groups. BoHV-4-A-PPRV-H-ΔTK immunization therefore elicited both CD4+ and CD8+ T cell responses against PPRV-H epitopes.

### BoHV-4-A-PPRV-H-ΔTK Immunization Stimulates Anti-PPRV Cytotoxic T Lymphocytes (CTL)

**Figure 4:**
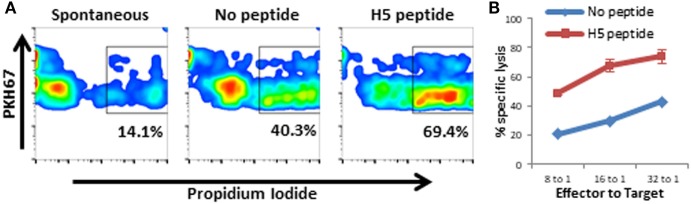
BoHV-4-A-PPRV-H-ΔTK vaccination induces cytotoxic T lymphocytes (CTL) specific for peste des petits ruminants virus hemagglutinin (PPRV-H) epitopes Splenocytes from BoHV-4-A-PPRV-H**-Δ**TK (BoHV-4-A-PPRV-H) immunized C57BL/6 mice were stimulated with H5 peptide from PPRV for 1 week *in vitro* and used as effector cells in flow cytometry-based cytotoxicity assays. RMA-s cell were used as target cells. RMA-s cell membrane was fluorescently labeled with PKH67 marker, and cells pulsed with peptide or left unpulsed as control. After incubation with effector cells, target cell lysis was evaluated by propidium iodide (PI) staining. **(A)** Target cell death was gated in bright PKH67 + and PI + event as shown. **(B)** Representative specific cytotoxicity of target cells pulsed with H5 peptide when cultured with H5-stimulated splenocytes is shown.

Splenocytes from BoHV-4-A-PPRV-H-ΔTK-immunized mice were H5 peptide-stimulated for 1 week *in vitro* and used as effector cells in flow cytometry-based cytotoxicity assays. RMA-s cells were used as target cells in these experiments. Splenocytes from BoHV-4-A-PPRV-H-ΔTK-immunized mice were capable of specifically lysing RMA-s cells pulsed with H5 peptide (Figures 4A,B). These data indicate that BoHV-4-A-PPRV-H-ΔTK immunization can promote CTL responses against PPRV infection.

### BoHV-4-A-PPRV-H-ΔTK Immunization Induces a Specific Neutralizing Antibody Response against PPRV-H

To determine the presence of neutralizing antibodies, sera from vaccinated mice obtained at 28 days post first immunization (7 days post booster inoculation) were assayed in a virus neutralization test. All BoHV-4-A-PPRV-H-ΔTK vaccinated animals showed PPRV-specific neutralizing antibodies with neutralization titers between 160 and 320 (Table 1).

**Table 1:** *In vitro* analysis of neutralization of Nigeria 75/1 peste des petits ruminants virus (PPRV) strain infectivity.

Neutralization titer^a^			
Source of antigen^b^	Mouse no.	0 DPI^c^	28 DPI^d^
PBS	1 to 10	<10	<10
BoHV-4A	1 to 10	<10	<10
BoHV-4-PPRV-H	1	<10	120
	2	<10	360
	3	<10	120
	4	<10	360
	5	<10	120
	6	<10	360
	7	<10	120
	8	<10	120
	9	<10	120
	10	<10	120

*^a^SN titers were determined against Nigeria 75/1 PPRV strain and expressed as the reciprocal of the last dilution of serum that neutralized 50% of the virus-specific cytopathic effect in flat bottom 96 plates*.

*^b^Intraperitoneally inoculations of different antigens (see Materials and Methods)*.

*^c^Analyzed sera obtained from uninfected mice*.

*^d^Analyzed sera obtained from infected mice 28 days post first immunization (7 days post-boost)*.

No neutralization activity was detected in pre-immune sera or sera from mice injected with either PBS or BoHV-4-A. These results show that *in vivo* inoculation of recombinant BoHV-4-A expressing the PPRV-H protein is able to induce the production of PPRV neutralizing antibodies, suggesting that this approach has the potential to confer protective immunity to BoHV-4-A-PPRV-H-ΔTK vaccinated animals.

## Discussion

In developing countries, most of the population is engaged in small-scale farming, 80% of these households keep livestock mostly constituted by small ruminants, primarily sheep and goats. Their productivity is constrained by multiple factors, including infectious diseases where PPR represents one of the most important ones. Vaccination can reduce animal mortality, increase milk and meat production, and positively impact on household revenues. As a result, vaccination also contributes to poverty alleviation by increasing household benefits and freeing income for food, healthcare, or child education. Therefore, new effective vaccines that target diseases that hamper farming in developing countries will have great social and economic benefit (33).

For PPRV eradication campaign, a DIVA vaccine would be of great value to facilitate PPRV sero-surveillance programs and speed up strategies for disease control and eradication (34). The most important drawback when a classical live attenuated vaccine is used is the inability to distinguish the immune response stimulated by vaccination from the one induced by a natural infection. A DIVA vaccine would therefore be a smart solution that combines vaccination with sero-surveillance. DIVA vaccine can be applied not only with gene-deleted marker vaccines (35) but also with sub-unit vaccines (36), heterologous vaccines (37), and recombinant vector-based vaccines. With regard to the last case and as an alternative viral vector, a BoHV-4-based vector platform was employed in the present work to deliver and express PPRV-H gene in transduced cells of immunocompetent mice as surrogate animal model. Although no murine model for PPRV induced disease exists, they represent an invaluable model to initially test the immunity induced by new prototype vaccines. The direct use of large animals could represent a major waste of resources, in terms of maintenance and biosafety containment structures, especially in the event of experiment failure. Data provided by immunized mice not only can be obtained quickly and cheaply but also they could represent a predictive and orientative tool of the vaccine immunogenicity in the natural host, e.g., goats and sheep in the specific case of PPRV (4, 25). PPRV-H protein possesses both hemagglutinin and neuraminidase activities and has a hydrophobic domain at the N-terminus (amino acid position 35–38), which remains within the mature protein acting as a signal peptide that anchors the protein into the membrane (38). The presence of N-terminal 34 amino acids located inside the membrane characterizes PPRV-H as a type II glycoprotein (38). Since PPRV-H protein has been shown to be a good candidate antigen (3, 4), a recombinant BoHV-4 delivering an optimized PPRV-H expression cassette was constructed in order to test the immunogenicity of this BoHV-4-based vector and exploit it as a DIVA vaccine platform for PPR vaccination. BoHV-4 has no clear direct disease association; however, its pathogenic potential cannot be absolutely excluded. This is an important consideration since it is to be used as a gene delivery vector. In fact, BoHV-4 has been often associated with postpartum metritis in cattle along with specific endometotropic (39, 40). The secretion of prostaglandin E2 (PGE2) and then stimulation of viral replication by PGE2, TNF-α, and lipopolysaccharide (LPS) were suggested as a pathogenic model for BoHV-4 and bacterial co-infection in endometritic cows (41–43). Therefore, a putative non-pathogenic biotype of BoHV-4 (BoHV-4-A) isolated from the milk cell fraction of a healthy cow whose genome was cloned as a bacterial artificial chromosome (pBAC-BoHV-4-A) (14) was employed. Importantly, BoHV-4-A-based vector behaves like a replicating incompetent viral vector in both wild-type and immunocompromised mice, showing complete absence of pathogenicity (16, 17, 19, 27, 44, 45). PPRV-H ORF was customized under the transcriptional control of the CMV promoter and integrated into BoHV-4-A genome TK locus. The derived replication-deficient recombinant vector could transduce mammalian cells and expressed PPRV-H protein. This construct could therefore potentially elicit immunity to the transgene. Genetic stability of viral vectors remains a very important issue, since recombinant viral vectors constitute “genetically modified organisms” (GMO). In our case, the BoHV-4-A-PPRV-H-ΔTK construct was stable through several passages. Relevant planning will however be needed before this recombinant vector can legally be licensed for employment in the field.

Protective natural immunity to morbilliviruses requires both humoral and cellular components of the adaptive immune system. Humoral immunity can protect against the prototype morbillivirus measles virus re-infection, whereas cellular immunity controls virus clearance and dissemination (46, 47). In the present work, mice immunized with BoHV-4-A-PPRV-H-ΔTK produced CD4+ and CD8+ T cell responses against PPRV-H epitopes and promoted CTL responses against PPRV. This recombinant vector vaccine can therefore potentially stimulate the T cell immunity essential for virus clearance. It will be interesting in future work to determine whether similarly to recombinant adenovirus vaccines (29), BoHV-4-A-PPRV-H-ΔTK immunization can trigger memory T cell responses in PPRV natural hosts.

However, the most striking results were related to the production of virus neutralizing antibodies (VNAs) against PPRV. It was previously shown that a neutralization titer higher than 10 correlates with a long-lasting humoral response and could be considered as a successful vaccination and protection indicator in the field (48, 49). In this pilot study, the lowest VNA titer obtained for all vaccinated mice was never below 120. It could thus be speculated that vaccinated BoHV-4-A-PPRV-H-ΔTK animals could be protected from virulent PPRV challenge when this protocol will be/is applied in the natural host. This is further supported by the fact that BoHV-4 has been successfully used in sheep and goats (13, 18). BoHV-4-based vector delivering H alone also induced neutralization titers higher than those obtained with other viral vectors delivering both H and F antigens, which is in line with the concept that H glycoprotein of Paramyxovirus is a stronger inducer of VNA than the F glycoprotein (50, 51). Despite the notion that antibody immune response against PPRV is the main factor for an efficient protection, cellular immune response can be also important for virus clearance. In some cases, protection has been obtained even with undetectable level of VNA titers (52, 53). The high VNA titer levels and the induction of cellular immunity after BoHV-4-A-PPRV-H-ΔTK immunization indicate that this recombinant vector vaccine has the potential to protect from virulent viral challenge. The induction of humoral and cellular immunity after BoHV-4-A-PPRV-H-ΔTK inoculation indicates that this vaccine can trigger PPRV immunity in the natural host both in an experimental setting and in the field.

In conclusion, in the present paper, it was demonstrated that BoHV-4-A-PPRV-H-ΔTK is able to induce a strong specific immune response against PPRV. These findings are paving the way for BoHV-4-A-PPRV-H-ΔTK use as a safe, large, potent, non-integrative, replicating competent viral vector for PPR vaccination and eradication.

## Availability of Data and Material

Available upon request.

## Ethics Statement

Experiments were performed in a disease-secure isolation facility (BSL3) at the Centro de Investigación en Sanidad Animal (CISA), in strict accordance with the recommendations of the Code for Methods and Welfare Considerations in Behavioral Research with Animals (Directive 86/609EC; RD1201/2005). Experiments were approved by the Committee on the Ethics of Animal Experiments (CEEA) of the Spanish Instituto Nacional de Investigación y Tecnología Agraria y Alimentaria (INIA) and the “Comisión de ética estatal de bienestar animal.”

## Author Contributions

GD conceived the experiments. VF, JR, AV, GT, NS, FM, VM, CP, and GD performed the experiments. GD, LL, SC, JR, VM, and SO analyzed the data. GD wrote the paper.

## Conflict of Interest Statement

The authors declare that the research was conducted in the absence of any commercial or financial relationships that could be construed as a potential conflict of interest.

## Abbreviations

BoHV-4, bovine herpesvirus 4; BAC, bacterial artificial chromosome; BEK, bovine embryo kidney; HEK293T, human embryo kidney 293 T; VDS, vero dog-SLAM; FBS, fetal bovine serum; EMEM, eagle’s minimal essential medium; DMEM, dulbecco’s modified essential medium; DMSO, dimethyl sulfoxide; MOI, multiplicity of infection; TCID50, tissue culture infectious doses 50; EDTA, ethylenedinitrilo-tetraacetic; PEI, polyethylenimine; CPE, cytopathic effect; MFI, mean fluorescence intensity; PPR, peste des petits ruminants; PPRV, peste des petits ruminants virus; PPRV-H, peste des petits ruminants virus hemagglutinin; H, hemagglutinin; DIVA, differentiating infected from vaccinated animals; OIE, office international des epizooties.
